# PRELIMINARY FEASIBILITY, UTILITY, AND ACCEPTABILITY OF THE TBI-AD/ADRD CAREGIVER SUPPORT INTERVENTION (TACSI)

**DOI:** 10.1093/geroni/igad104.2552

**Published:** 2023-12-21

**Authors:** Robyn Birkeland, Elizabeth Albers, Katie Louwagie, Edward Ratner, Jacob Finn, Sherry Chesak, Joseph Gaugler

**Affiliations:** University of Minnesota, Minneapolis, Minnesota, United States; University of Minnesota School of Public Health, Minneapolis, Minnesota, United States; University of Minnesota, Minneapolis, Minnesota, United States; Minneapolis VA Health Care System, Minneapolis, Minnesota, United States; Minneapolis VAHCS, Minneapolis, Minnesota, United States; Mayo Clinic, Rochester, Minnesota, United States; University of Minnesota, Minneapolis, Minnesota, United States

## Abstract

Research has established that dementia care partners may encounter socio-emotional and physical health concerns as a consequence of providing dementia care. Similarly, care partners for people living with Traumatic Brain Injury (TBI) often experience a range of stressors and negative mental health outcomes due to their care demands. This study aims to support the unique subset of family care partners facing the challenge of caring for relatives who have both diseases, Alzheimer’s Disease/Alzheimer’s Disease related dementia (AD/ADRD) and a history of TBI. Phase I of this study focused on the design and subsequent refinement of the TBI-AD/ADRD Caregiver Support Intervention (TACSI). TACSI is a six-session telehealth intervention providing tailored psychosocial and psychoeducational coaching. Session topics include education on TBI and dementia, stress management, lifestyle planning, and communication skills. In partnership with the Minneapolis VA and the Mayo Clinic-Rochester, we enrolled 15 care partners for Phase I. Mixed methods data from 3-month follow-up surveys and qualitative data from post-intervention interviews established the feasibility, utility, and acceptance of TACSI. Care partners found the program valuable and would recommend it to others. They reported that TACSI helped with their stress management. Some participants shared that TACSI would provide greater benefit to newer caregivers needing more education on the diseases and/or caregiving. Phase I feedback will be applied to improve TACSI content and delivery for the Phase II randomized controlled trial evaluation. TACSI Phase I utility, acceptance, and feasibility outcomes will be highlighted as well as next steps for evaluation.

